# Supplementary material for: Dehumanization as a Response to Uncivil and Immoral Behaviors
Source: Eur J Investig Health Psychol Educ. 2022 Sep 15;12(9):1415–26. doi: 10.3390/ejihpe12090098 (PMC9497610; doi:10.3390/ejihpe12090098)
Supplement: Supplementary file 1 [file ejihpe-12-00098-s001.zip › ejihpe-1889568-supplementary.pdf]

Supplementary Material

Pretest study of 120 civil and uncivil behaviors (Study 1) 2

Table S1 10

Table S2 11

Table S3 16

Behaviors selection (Study 1) 17

Traits selection (Study 1) 19

Behaviors selection (Study 2) 20

Table S4 21

Table S5 22

Table S6 23

Table S7 24

Pretest study of 120 civil and uncivil behaviors (Study 1)

The aim of this pre-test was to obtain a comprehensive database of civil and uncivil behaviors and provide information about how such behaviors are perceived in a series of dimensions related to civility and humanness. Besides, we were interested in analysing how these perceptions are interrelated. Specifically, our goal was to explore the extent to which individuals believe that civil and uncivil behaviors are related to the humanness of behavior, provide moral information, have social repercussions, are related with universal nature and are frequent. The results of this first study provided us with many evaluations of how civil and uncivil behaviors are perceived in humanness and other relevant dimensions to civility. These findings will be useful to dehumanization researchers as well as to social cognition researchers more generally.

Method

Participants

A total of 360 university students, all residents in Spain, participated in this study. Participants' ages ranged from 17 to 51 (M = 20.01; SD = 3.46); 261 were female. They were divided into 12 samples of 30 participants each. The group was made up of students of Psychology (n = 293), Labor Relations (n = 9), Nursing (n = 7), and Physiotherapy (n = 51). Each participant evaluated ten behaviors in eight relevant dimensions.

Materials and procedure

Pilot study

The behavioral descriptions were obtained from pilot questionnaires, personal interviews, and group meetings in which 50 individuals similar to those of the study sample in age and studies were asked to name behaviors that they considered civil and

uncivil with respect to other people, the surroundings, or street furniture. Based on these instructions, more than 200 contributions were received listing those behaviors that are considered respectful and those that are found to be disruptive to society.

Among the 200 behaviors, many of them repeated ideas, for example: "leave garbage in the street," "throw paper on the street," "leave trash after having a picnic," "leave paper in the field," "leave paper and trash in public places." The repeated behaviors were grouped together to create a single category, in this example: "Throwing paper and trash on the street." In addition, incivilities include everyday experience that are not considered as behaviors that violate formal legal codes, for this reason, behaviors which represented clear infractions of the law or criminal acts were eliminated. Finally, the descriptions were shortened to make them easier to manage within the experimental designs, as overly long utterances require a lot of time to process. A total of 45 civil behaviors and 75 uncivil behaviors were obtained. Some of the uncivil behaviors referred to relatively innocuous actions that are harmless yet annoying (e.g., laughing loudly in public), show a lack of courtesy (e.g., interrupting someone while they are talking), or reflect a lack of personal hygiene and manners (e.g., body odor, long or dirty fingernails). Others constituted clear violations of social norms that are punishable by local bylaws (e.g., not cleaning up after your dog, or parking in a spot reserved for the disabled).

The civil behaviors, for their part, represented a broad spectrum of behaviors, some of which reflect conformity with norms (e.g., using the crosswalk, taking out the garbage at the established time) or have altruistic ends (e.g., giving your seat to an elderly person). Here too, there were some behaviors that had to do with personal hygiene in social interactions (e.g., covering your mouth when you cough or sneeze) and others referring to acts of courtesy (e.g., holding the door open for someone when entering or leaving a room).

Main study

The 120 behaviors were distributed into 12 questionnaires, with care taken to ensure that each contained a similar proportion of descriptions of civil and uncivil behaviors. Each of these descriptions was evaluated along the eight dimensions relevant to this study. These dimensions were drawn from studies on civility and humanness. Specifically, the dimensions of "Valence," "Frequency," and "Universality" were adapted from the studies of Haslam et al. (2005), Haslam, Bastian, and Bissett (2004) and Wilson and Haslam (2012). The "Moral information" dimension was drawn from studies on emotional terms associated with infrahumanization (Demoulin et al., 2004). The dimensions of "Social repercussions," "Censure," and "Civility" were adapted from studies by Brauer and Chaurand (2008, 2009). Finally, the dimension of "Humanness" was inspired in the measure Ascent of Man (Kteily et al., 2015). The question consisted of a description of the idea of evolution, in order to highlight a salient distinction between early human ancestors and modern humans; that is, the full realization of cognitive ability and cultural expression.

The following questions were presented to the participants, using the wording below:

1. *Valence.* "Please indicate for each of the behaviors below the extent to which you find it positive or negative." Five options were then presented, ranging from 1 (Negative) to 5 (Positive).

2. *Frequency.* "In your opinion, how often do you think people exhibit each of the following behaviors?" Five intervals were then presented, ranging from 1 (Not often at all) to 5 (Very often).

3. *Moral information on the agent of the behavior.* “There are some behaviors that, when we see them being exhibited, give us a lot of information about the nature and moral quality of the person exhibiting them. In other cases, these behaviors give us very little information. Please indicate the extent to which each behavior gives us information about the morality of the person exhibiting it.” Five options were then presented, ranging from 1 (No moral information) to 5 (Much moral information).

4. *Social repercussion.* “Some of our behaviors also have consequences for those around us. To what extent do you think that the following behaviors have repercussions on other people?” The five options presented ranged from 1 (Few repercussions) to 5 (Many repercussions).

5. *Censure of the behavior.* “When certain behaviors are observed, people may react more or less intensely to those exhibiting them. In some cases, these reactions are positive (they are praised, applauded, etc.) and in others they are negative (they are reproached, criticized, etc.). In other cases, there is no reaction. To what extent do you believe that the following behaviors provoke intense public reactions?” Five options were then presented, ranging from 1 (Provokes no reaction) to 5 (Provokes intense reaction).

6. *Humanness.* “Although humans are more highly evolved than animals, we sometimes exhibit behaviors that are more animal than human. This happens regardless of the technological and modern context in which we find ourselves. In other words, we can observe people exhibiting behaviors involving modern artifacts (mobile phones, cars, etc.) that, at the same time, are more similar to animal behavior than what you would expect from highly evolved humans. To what extent are the following behaviors consistent with the evolutionary level attained by humans? Five options were presented, ranging from 1 (Not very consistent) to 5 (Very consistent).

7. *Universality.* “Indicate on a scale from 1 to 5 the extent to which you believe that the following behaviors are exhibited solely by people of your own culture, or by people of some cultures, or by people of all cultures. Here, five options were presented, ranging from 1 (Only my own culture) to 5 (All cultures).

8. *Civility*. “Finally, please respond to the following question: To what extent do you believe that the following behaviors are more or less civil? Please consider the term “civil” to refer to a type of ethical behavior that includes courtesy, manners, good citizenship, and concern for the wellbeing of the members of our community.” Five options were then presented, ranging from 1 (Uncivil) to 5 (Civil).

The research was presented as a study of the way people reacted to behaviors related to urban sustainability and social coexistence. The participants were shown each question followed by the ten behavioral descriptions and asked to score each on the scale provided. The questionnaires were administered in the students’ classrooms, once informed consent had been received and data on the participants’ sex, age, and university studies had been recorded.

Results

Reliability

First, the reliability of the scores was determined by means of inter-rater reliability analysis and it was verified that the mean scores of the civil behavior descriptions differed significantly from the uncivil behavior descriptions in the civility dimension. To determine whether participants were able to reliably evaluate behaviors in all eight dimensions, the procedure established by Rothbart and Park (1986) was followed. Specifically, in each sample, the mean was calculated for each of the eight dimensions in the ten behavioral descriptions, and then the bivariate correlation was calculated between

these means and the scores awarded by each participant in the 80 responses. Given the high correlation observed between the judgments of each participant and the sample mean, we decided to exclude 25 participants whose correlations were not significant (p < .05). Therefore, the number of participants in each sample ranged from 25 to 29 (see Table S1).

Then, reliability was calculated using Cronbach's alpha and the intraclass correlation coefficient. Although this procedure usually analyzes the degree of intercorrelation between items, in this case, with the transposed matrix, what the analysis showed us was the degree of inter-rater agreement in each of the 80 items (ten behaviors and eight items).

Once the reliability of the judgments provided by the participants of the different samples had been confirmed, the mean scores of the 120 behaviors in the eight dimensions were calculated and transferred to a new matrix (see Table S2).

Comparison between civil and uncivil behaviors

To further examine whether behaviors cluster together because of their civility and humanness ratings, a cluster analysis was run on the 120 behaviors using their civility and humanness ratings. A hierarchical cluster analysis using Ward's method produced two clusters. Cluster 1 (42 behaviors) was defined by behaviors that reflect civility, whereas Cluster 2 (78 behaviors) was defined by incivilities.

There were significant differences between clusters in terms of civility, $t(111) = 30.88$, $p < .001$; humanness, $t(118) = 21.42$, $p < .001$; frequency, $t(118) = -2.24$ $p < .05$; universality, $t(118) = 3.46$, $p < .001$; censure of the behavior, $t(118) = -3.75$, $p < .001$; valence, $t(118) = 24.99$, $p < .001$; and moral information, $t(118) = 2.82$, $p < .01$. There were no differences between the clusters in terms of social repercussion, $t(105) = 0.748$,

$p > .05$. Table S2 summarises the means and standard deviations of the clusters in each dimension.

Correlational analysis

The correlations among the conceptual judgments are presented in Table S3. For civil behaviors, civility was positively and strongly associated with positivity ($r = .78$, $p < .001$), moral information ($r = .72$, $p < .001$), and humanness ($r = .69$, $p < .001$). Civility was also positively associated with universality ($r = .50$, $p < .001$), social repercussion ($r = .56$, $p < .001$), and social reactions ($r = .40$, $p < .001$). Frequency was not correlated with civility ($r = .10$, *n.s.*).

In the case of uncivil behaviors, civility was positively and strongly associated with valence ($r = .88$, $p < .001$; the less civil the behavior perceived, the less positive valence can be extracted) and humanness ($r = .53$, $p < .001$; the less civil the behavior perceived, the less humanness is attributed) and negatively associated with moral information ($r = -.69$, $p < .001$; the less civil the behavior perceived, the more moral information about the agent can be extracted), social repercussion ($r = -.70$, $p < .001$; the less civil the behavior perceived, the more social repercussion is attributed), and social reactions ($r = -.47$, $p < .001$; the less civil the behavior perceived, the more social reaction it displays). Civility was not positively associated with universality ($r = .02$, *n.s.*) or frequency ($r = -.12$, *n.s.*) in uncivil behaviors.

Conclusions

The aim of this pretest was to obtain a comprehensive database of civil and uncivil behaviors and provide information about how such behaviors are perceived in a series of relevant dimensions related to civility and humanness and how these perceptions are

interrelated. The result is a list of 120 behaviors in the civil domain that can be used across research paradigms to eliminate confounds and the need for repeated, costly pretesting.

Specifically, our findings indicate that there is a strong relationship between civility and humanness. Correlational analysis confirmed that both civil and uncivil behaviors are associated with social reactions, morality, and social repercussion. Valence and humanness are closely related with civility, showing that the more civil the behavior is perceived to be, the more positive and human it is considered to be. Besides, civil and uncivil behaviors are characterized by distinct conceptual profiles. Specifically, universality is only associated with civil behaviors. This finding seems to indicate that civil behaviors are perceived as more universal, whereas uncivil behaviors are considered more cultural-dependent. In this sense and considering the theoretical framework that highlights that civility is based on conventional norms (Kingwell, 1995), our results seems to indicate that uncivil behaviors are considered more conventional than civil behaviors. This result could lead to different social expectations towards civil and uncivil behaviors, and even more tolerance to accept uncivil behaviors since they are partially explained as an expression of cultural conventions. Future studies could help to explore this assumption.

In short, this pretest confirmed the association between the dimensions of civility and humanness, as well as the association between civility and valence, and civility and moral information.

Table S1

Sample size before and after correlation analysis and Cronbach's alpha.

Sample	Original N	Final N	Cronbach's alpha
A	30	27	.958
B	30	28	.879
C	30	28	.951
D	30	29	.908
E	30	29	.951
F	30	28	.943
G	30	29	.948
H	30	29	.948
I	30	26	.954
J	30	29	.941
K	30	25	.964
L	30	28	.962
TOTAL	360	335	

Table S2

Mean scores and standard deviation of 120 civil and uncivil behaviors in eight dimensions related to the perception of humanness.

	Frequency	Universality	Censure	Valence	Moral Information	Humanness	Social repercussion	Civism
	M (SD)	*M (SD)*	*M (SD)*	*M (SD)*	*M (SD)*	*M (SD)*	*M (SD)*	*M (SD)*
**Giving your seat up to an older person** (Ceder el asiento a una persona mayor)	3,26 (0,81)	3,85 (0,66)	3,22 (0,93)	4,81 (0,48)	4,67 (0,48)	4,59 (0,57)	4,30 (0,78)	5,00 (0,00)
**Offering help to those who need it** (Ofrecer ayuda a quien lo necesita)	3,07 (0,60)	4,04 (0,79)	3,71 (1,05)	4,79 (0,79)	4,79 (0,63)	4,32 (0,98)	4,54 (0,88)	4,89 (0,57)
**Helping push a broken-down car** (Ayudar a empujar un coche averiado)	2,60 (1,08)	3,58 (1,14)	3,65 (1,20)	4,73 (0,72)	4,58 (0,64)	4,27 (1,04)	4,38 (0,90)	4,84 (0,37)
**Holding your cigarette away so as not to disturb others with the smoke** (Alejar el cigarro para no molestar con el humo)	2,52 (1,06)	3,52 (0,83)	3,28 (1,00)	4,76 (0,79)	4,45 (0,74)	4,31 (0,97)	4,21 (1,11)	4,78 (0,51)
**Taking care of urban furniture** (Cuidar el mobiliario urbano)	2,41 (0,82)	3,76 (0,87)	2,79 (1,15)	4,83 (0,47)	4,24 (0,79)	4,25 (1,00)	4,22 (0,85)	4,74 (0,81)
**Picking up something that another person has dropped** (Recoger del suelo algo que se le ha caído a otra persona)	2,69 (1,20)	4,03 (0,78)	3,10 (1,05)	4,46 (0,79)	4,59 (0,95)	4,07 (1,10)	3,93 (1,05)	4,74 (0,59)
**Saying please when asking for things** (Pedir las cosas por favor)	3,55 (0,83)	3,72 (1,03)	3,55 (0,95)	4,72 (0,65)	4,69 (0,81)	4,55 (1,02)	4,38 (0,90)	4,69 (0,71)
**Offering to carry a heavy bag for an old woman** (Ofrecerse a llevar una bolsa pesada a una anciana)	1,93 (0,80)	3,29 (0,81)	3,55 (0,99)	4,50 (0,64)	4,31 (0,97)	4,24 (0,91)	3,93 (1,12)	4,66 (0,55)
**Respecting the turn in a queue** (Respetar el turno en una cola)	3,88 (0,99)	3,78 (0,80)	3,37 (1,31)	4,19 (0,92)	3,96 (1,06)	4,07 (0,73)	3,74 (1,06)	4,59 (0,50)
**Waiting your turn in conversation** (Respetar el turno de palabra)	3,07 (0,94)	3,54 (0,96)	3,39 (1,03)	4,46 (0,58)	4,18 (0,77)	4,36 (0,91)	3,75 (0,93)	4,54 (0,74)
**Giving thanks when someone does you a favor** (Dar las gracias cuando alguien te hace un favor)	3,78 (0,93)	4,04 (0,98)	3,18 (1,09)	4,50 (0,51)	3,96 (0,92)	4,21 (0,83)	3,50 (1,00)	4,50 (0,88)
**Recycling glass** (Reciclar el vidrio)	2,24 (0,91)	3,14 (1,03)	2,41 (1,24)	3,97 (1,38)	3,97 (1,12)	4,38 (1,08)	4,24 (1,15)	4,48 (0,95)
**Yielding on the road** (Ceder el paso en la carretera)	3,04 (0,93)	3,38 (0,92)	3,35 (1,20)	4,31 (0,97)	4,00 (0,98)	4,12 (1,07)	3,81 (1,13)	4,48 (0,92)
**Flushing the toilet after using a public washroom** (Tirar de la cadena después de usar un baño público)	3,17 (1,10)	4,03 (0,87)	3,41 (1,24)	4,10 (1,37)	3,79 (0,98)	4,07 (0,81)	3,89 (1,29)	4,44 (0,85)
**Giving way when entering or leaving a room** (Ceder el paso al entrar o salir)	3,25 (0,89)	3,75 (0,89)	3,25 (0,89)	4,29 (0,66)	3,93 (0,90)	4,18 (0,82)	3,50 (0,84)	4,43 (0,88)
**Washing your hands after going to the bathroom** (Lavarse las manos después de ir al baño)	2,71 (1,12)	3,68 (1,12)	2,21 (1,07)	4,32 (0,94)	2,86 (1,43)	4,11 (1,17)	2,93 (1,27)	4,39 (0,92)
**Throwing your chewing gum away in a tissue** (Tirar el chicle en un pañuelo)	2,90 (1,32)	3,71 (1,08)	2,10 (1,01)	4,21 (0,77)	3,55 (1,35)	4,23 (0,71)	3,07 (1,30)	4,33 (0,68)
**Smelling good** (Oler bien)	3,55 (0,78)	3,59 (1,05)	4,17 (0,93)	4,52 (0,78)	3,62 (1,52)	4,21 (1,18)	3,90 (1,18)	4,31 (0,81)
**Giving your loose change to a beggar** (Dar una moneda a un mendigo)	2,48 (0,78)	3,59 (1,15)	2,83 (1,20)	4,24 (0,83)	4,14 (1,06)	4,19 (0,79)	3,59 (1,31)	4,30 (0,82)
**Not answering your mobile phone when you are with other people** (No atender al móvil cuando estás con otras personas)	2,15 (1,10)	3,37 (0,79)	3,11 (1,31)	4,48 (0,94)	4,19 (1,00)	3,89 (1,31)	3,52 (1,28)	4,30 (1,03)
**Avoiding make noise in public spaces** (Evitar hacer ruido en los espacios públicos)	2,59 (1,05)	3,24 (0,79)	2,69 (1,39)	4,14 (1,19)	3,34 (1,14)	3,76 (1,18)	3,52 (1,24)	4,28 (0,88)
**Not stepping on flowers in public gardens** (No pisar las flores en los jardines públicos)	2,79 (1,26)	3,21 (0,90)	2,21 (1,08)	3,93 (1,03)	3,28 (1,13)	3,34 (1,26)	2,66 (1,26)	4,25 (1,00)

**Picking up a piece of paper lying on the street** (Recoger un papel tirado en la calle)	1,44 (0,70)	3,22 (0,89)	2,30 (1,14)	4,07 (1,00)	4,04 (0,94)	3,96 (0,59)	3,04 (1,22)	4,22 (0,85)
**Not parking on the sidewalk** (No aparcar encima de la acera)	2,86 (1,19)	3,52 (0,91)	2,59 (1,15)	3,97 (1,02)	3,03 (1,02)	3,41 (1,09)	3,39 (1,29)	4,15 (0,91)
**Respecting the cycle lane** (Respetar el carril bici)	2,52 (0,99)	3,10 (0,94)	2,97 (0,98)	3,79 (1,18)	3,69 (1,28)	3,52 (1,09)	3,79 (1,15)	4,14 (1,27)
**Wearing clean and well-kept clothes** (Ir con ropa limpia y cuidada)	4,07 (0,90)	3,61 (1,20)	3,11 (1,42)	4,56 (0,70)	3,93 (1,30)	4,46 (0,88)	3,54 (1,45)	4,14 (1,04)
**Using the pedestrian crossing** (Cruzar por el paso de peatones)	3,59 (0,84)	3,85 (1,03)	1,89 (0,89)	3,96 (0,98)	2,78 (1,05)	3,59 (1,05)	3,15 (1,20)	4,12 (0,82)
**Covering your mouth with your hand when coughing or sneezing** (Taparse la boca con la mano al toser o estornudar)	3,26 (0,98)	3,50 (1,14)	2,65 (1,06)	3,38 (1,44)	2,88 (1,11)	3,59 (1,37)	3,19 (1,00)	4,11 (1,31)
**Throwing your cigarette butts in the rubbish** (Tirar las colillas en una papelera)	2,34 (1,17)	3,41 (1,05)	2,28 (1,07)	3,66 (1,32)	3,97 (1,02)	3,90 (1,18)	3,54 (1,07)	4,07 (1,27)
**Respecting parking places reserved for people with disabilities** (Respetar el parking para personas con discapacidad)	3,14 (1,30)	3,07 (1,07)	3,24 (1,38)	3,48 (1,68)	4,17 (1,17)	3,97 (1,21)	4,31 (1,17)	4,07 (1,58)
**Being punctual in appointments** (Ser puntual en las citas)	2,82 (0,86)	3,64 (0,95)	3,71 (1,08)	4,36 (1,03)	4,04 (0,88)	4,14 (0,97)	4,04 ((1,07)	4,04 (1,32)
**Serving food to others before yourself** (Servir la comida a los demás antes que a sí mismo)	3,59 (0,93)	3,31 (1,05)	2,30 (0,91)	3,77 (1,14)	3,50 (1,07)	3,78 (1,12)	2,52 (1,01)	3,96 (1,22)
**Not putting your feet up on the seat** (No poner los pies encima de otro asiento)	2,71 (1,12)	3,55 (1,12)	2,57 (1,07)	3,52 (1,18)	3,14 (1,22)	3,36 (1,10)	3,19 (1,30)	3,96 (1,08)
**Having good breath** (Tener buen aliento)	3,48 (0,83)	3,86 (0,83)	3,28 (1,16)	4,48 (0,57)	2,66 (1,29)	3,90 (1,05)	3,79 (0,94)	3,93 (1,03)
**Not smoking while others are eating** (No fumar mientras otros están comiendo)	2,85 (0,99)	3,50 (0,95)	2,59 (0,89)	3,92 (1,13)	3,26 (1,10)	3,96 (1,06)	3,00 (1,18)	3,93 (1,00)
**Turning off your mobile phone in the cinema** (Desconectar el móvil en el cine)	2,18 (1,19)	3,59 (0,93)	2,96 (1,29)	4,04 (1,07)	3,11 (1,26)	4,07 (0,98)	3,86 (0,85)	3,89 (1,29)
**Picking up dog droppings** (Recoger los excrementos del perro)	2,92 (0,97)	3,28 (1,24)	4,12 (0,95)	3,85 (1,78)	4,27 (0,83)	3,73 (1,43)	4,19 (1,13)	3,88 (1,69)
**Covering your mouth when you yawn** (Bostezar tapándose la boca)	3,48 (1,06)	3,38 (0,98)	2,55 (1,27)	3,93 (0,84)	3,38 (1,27)	3,86 (0,92)	2,52 (1,12)	3,86 (0,99)
**Not starting to eat until everyone is served** (No empezar a comer antes de que todos estén servidos)	2,93 (1,00)	2,77 (0,95)	2,23 (0,82)	3,27 (1,15)	3,20 (1,12)	3,63 (1,21)	2,22 (1,01)	3,85 (1,29)
**Warning of dripping from a tap in a public washroom** (Avisar del goteo de un grifo en un baño público)	1,61 (0,96)	3,46 (1,20)	2,29 (1,24)	3,64 (0,78)	3,32 (1,56)	3,64 (1,03)	2,82 (1,19)	3,82 (1,09)
**Taking out the garbage at the set time** (Sacar la basura a la hora establecida)	1,86 (1,06)	3,17 (1,07)	1,69 (0,93)	3,34 (0,77)	2,83 (1,04)	3,28 (1,13)	2,79 (1,26)	3,72 (0,96)
**Respecting the no-parking signs in front of garages** (Respetar los vados permanentes de los garajes)	2,93 (1,15)	3,21 (1,10)	3,26 (1,48)	3,86 (1,27)	3,50 (0,88)	3,86 (1,01)	4,18 (0,98)	3,68 (1,59)
**Having well-groomed nails** (Tener las uñas bien cuidadas)	2,93 (0,96)	3,38 (0,78)	2,55 (0,99)	3,28 (1,13)	2,66 (0,86)	3,24 (1,06)	2,31 (1,00)	3,25 (0,84)
**Returning extra change to a supermarket cashier** (Devolver una vuelta equivocada al cajero de un supermercado)	2,33 (0,96)	3,19 (1,00)	3,15 (1,29)	3,11 (1,40)	3,85 (1,06)	3,74 (0,98)	3,30 (1,14)	3,00 (1,41)
**Spitting into a handkerchief** (Escupir en un pañuelo)	1,82 (0,94)	3,25 (1,00)	3,00 (1,15)	2,61 (0,92)	3,14 (1,08)	3,00 (1,19)	2,71 (1,05)	2,93 (1,15)
**Washing your car on the street** (Lavar el coche en la calle)	4,32 (8,39)	2,79 (1,10)	1,81 (0,98	3,08 (0,57)	1,77 (0,86)	3,04 (0,96)	2,15 (0,83)	2,92 (0,49)
**Stretching in class or in a meeting** (Estirarse en clase o en una reunión)	3,93 (1,21)	3,44 (1,31)	2,74 (1,16)	2,67 (0,83)	2,30 (1,07)	2,70 (1,14)	2,15 (1,23)	2,85 (0,99)
**Holding a toothpick between your teeth** (Llevar un palillo de dientes entre los dientes)	1,40 (0,65)	2,46 (0,98)	1,69 (1,12)	2,62 (0,70)	1,65 (0,89)	2,42 (0,86)	1,58 (0,86)	2,68 (0,90)

**Copying the answers in an exam** (Copiarse en un examen)	3,85 (1,10)	4,19 (0,96)	3,04 (1,09)	2,48 (0,70)	2,89 (0,93)	3,12 (0,86)	2,78 (1,01)	2,67 (0,68)
**Licking food from your fingers** (Chuparse los dedos con restos de comida)	3,59 (1,05)	3,44 (1,25)	2,30 (0,95)	2,96 (0,65)	2,04 (0,85)	2,33 (1,11)	1,89 (1,09)	2,67 (0,83)
**Getting close to the other person when talking** (Acercarse mucho al otro al hablar)	2,59 (0,87)	2,66 (0,94)	3,90 (0,86)	2,34 (1,04)	2,97 (1,12)	3,00 (1,10)	3,86 (0,88)	2,62 (0,90)
**Laughing very loudly in public** (Reírse muy alto en público)	3,66 (0,97)	3,10 (1,05)	3,52 (0,95)	2,66 (1,01)	3,10 (1,11)	2,66 (0,77)	2,86 (1,13)	2,41 (0,63)
**Blowing your nose noisily in front of people** (Sonarse ruidosamente delante de la gente)	3,10 (1,11)	3,28 (0,92)	3,55 (1,06)	1,93 (0,80)	2,86 (1,30)	2,69 (0,97)	2,90 (1,08)	2,38 (0,98)
**Eating with your hands** (Comer con las manos)	3,25 (1,29)	3,93 (0,90)	2,82 (1,25)	2,64 (0,87)	2,43 (0,96)	2,25 (1,04)	2,54 (1,10)	2,36 (0,91)
**Using a friend to get a public benefit** (Recurrir a algún amigo para conseguir un beneficio público)	3,70 (0,82)	3,63 (0,97)	3,30 (1,07)	2,26 (0,76)	3,56 (0,80)	2,96 (1,13)	3,48 (0,70)	2,33 (0,83)
**Yawning loudly in public** (Bostezar fuerte en público)	3,00 (1,13)	3,28 (1,00)	2,76 (1,21)	2,55 (0,74)	2,62 (1,12)	2,26 (0,81)	2,44 (1,15)	2,30 (0,78)
**Interrupting another when he/she is speaking** (Interrumpir a otro cuando está hablando)	3,93 (1,04)	3,52 (1,16)	3,62 (0,90)	2,04 (1,02)	3,37 (1,18)	2,48 (1,12)	3,70 (0,87)	2,30 (1,10)
**Making inscriptions on trees** (Hacer inscripciones en los árboles)	3,00 (1,28)	3,34 (0,97)	2,28 (1,22)	2,24 (0,91)	2,52 (1,21)	2,41 (0,98)	2,31 (1,26)	2,28 (0,80)
**Having bad breath due to lack of hygiene** (Tener mal aliento por falta de higiene)	2,76 (1,18)	3,59 (0,87)	3,79 (1,05)	1,55 (0,74)	2,79 (1,05)	2,21 (1,01)	3,76 (0,95)	2,25 (0,84)
**Not informing of the dripping of a tap in a public washroom** (No avisar del goteo de un grifo en un baño público)	3,93 (1,60)	3,62 (1,15)	1,59 (0,98)	2,10 (0,77)	2,93 (1,31)	2,21 (1,08)	3,38 (1,12)	2,24 (1,02)
**Bathing in a public fountain** (Bañarse en una fuente pública)	1,31 (0,47)	3,38 (1,08)	4,07 (1,03)	2,14 (0,74)	3,17 (1,10)	2,04 (0,79)	3,04 (1,11)	2,22 (1,09)
**Rummaging in your ears with your finger** (Hurgarse los oídos con el dedo)	3,03 (1,30)	3,45 (1,02)	3,24 (1,27)	1,93 (0,84)	2,14 (1,16)	2,10 (0,86)	2,17 (1,20)	2,14 (0,83)
**Wearing dirty clothes** (Ir con la ropa sucia)	1,83 (0,71)	3,38 (1,01)	3,93 (0,84)	1,72 (0,80)	3,28 (1,33)	2,00 (0,90)	3,19 (1,33)	2,11 (0,85)
**Simulating an illness to avoid going to work** (Simular una enfermedad para no ir a trabajar)	3,26 (0,90)	2,58 (1,33)	2,63 (1,01)	1,96 (0,72)	3,58 (1,17)	2,89 (1,34)	3,11 (0,70)	2,11 (0,70)
**Making noise when chewing** (Hacer ruido al masticar)	3,14 (1,09)	3,48 (1,15)	3,07 (1,22)	2,21 (0,68)	2,69 (1,17)	2,17 (0,97)	2,39 (0,96)	2,11 (0,70)
**Showing up late for appointments** (Ser impuntual en las citas)	3,59 (0,82)	3,00 (0,96)	3,83 (0,85)	1,97 (1,05)	3,31 (0,93)	2,97 (0,98)	4,14 (0,92)	2,11 (1,03)
**Standing too close in queues** (Pegarse mucho al de delante en una cola)	3,88 (1,01)	3,46 (1,02)	3,58 (1,03)	1,73 (0,72)	3,12 (1,11)	2,38 (0,98)	3,85 (1,01)	2,08 (0,70)
**Smelling of sweat** (Oler mucho a sudor)	2,63 (0,88)	3,89 (0,80)	4,07 (1,04)	1,52 (0,58)	2,15 (1,29)	2,08 (0,84)	3,59 (0,93)	2,07 (0,73)
**Speaking with your mouth full** (Hablar con la boca llena)	2,85 (0,77)	3,32 (1,19)	3,61 (0,74)	1,70 (0,61)	3,46 (0,92)	1,79 (0,88)	2,89 (0,92)	2,07 (1,21)
**Wearing long and dirty nails (**Llevar las uñas largas y sucias)	2,00 (1,04)	3,48 (1,23)	3,31 (1,44)	1,58 (0,76)	3,08 (1,49)	1,69 (0,79)	2,65 (1,38)	2,04 (0,89)
**Picking your nose** (Meterse los dedos en la nariz)	2,55 (1,06)	3,89 (0,92)	3,41 (1,12)	1,86 (0,79)	2,28 (1,03)	1,93 (0,92)	2,00 (0,96)	2,04 (0,94)
**Not giving way when entering or leaving a room** (No ceder el paso al entrar o salir)	2,97 (0,98)	3,34 (1,17)	3,76 (1,02)	1,48 (0,83)	3,97 (1,15)	2,46 (1,04)	3,89 (1,28)	2,04 (1,51)
**Calling late at night** (Llamar por teléfono a altas horas de la noche)	2,83 (1,28)	3,14 (1,06)	3,55 (1,06)	2,55 (0,83)	2,66 (0,81)	2,86 (1,09)	3,59 (0,98)	2,04 (0,84)
**Not greeting a neighbor** (No saludar a un vecino de escalera)	2,36 (1,04)	2,92 (0,86)	2,65 (1,13)	2,04 (0,87)	3,85 (1,12)	1,96 (1,00)	3,28 (1,10)	2,00 (1,08)

**Not washing your hands after going to the bathroom** (No lavarse las manos después de ir al baño)	3,52 (1,12)	3,29 (0,94)	2,74 (1,29)	2,04 (0,88)	3,14 (1,04)	2,07 (1,25)	3,07 (1,15)	1,96 (0,88)
**Eating with dirty hands** (Comer con las manos sucias)	3,48 (1,24)	3,21 (0,82)	3,14 (1,09)	1,83 (0,85)	3,00 (1,16)	1,93 (1,07)	2,48 (1,35)	1,96 (1,15)
**Jumping the queue** (Colarse en una cola)	3,29 (1,12)	3,29 (1,24)	4,00 (1,12)	1,68 (0,90)	3,14 (0,80)	3,11 (1,13)	3,00 (0,98)	1,89 (1,41)
**Parking on the sidewalk** (Aparcar encima de la acera)	3,36 (0,91)	3,43 (1,14)	3,18 (1,09)	2,14 (0,89)	3,07 (1,09)	2,54 (1,07)	4,14 (0,85)	1,89 (0,84)
**Using privileged information to get something** (Usar información privilegiada para conseguir algo)	3,81 (1,00)	3,64 (0,99)	3,93 (1,04)	2,00 (0,98)	3,88 (0,91)	2,85 (1,35)	3,96 (0,94)	1,89 (1,59)
**Pretending that you are suffering to get something** (Aparentar que se está sufriendo para conseguir algo)	3,07 (1,00)	3,63 (0,97)	3,96 (0,94)	1,44 (0,70)	4,19 (0,92)	2,26 (1,10)	4,11 (0,80)	1,85 (0,96)
**Putting your feet up on the seat** (Poner los pies encima de otro asiento)	3,21 (0,96)	3,07 (1,21)	2,93 (1,09)	2,04 (0,79)	2,93 (1,09)	2,48 (0,89)	2,71 (0,94)	1,85 (1,09)
**Parking in a parking space reserved for people with disabilities** (Aparcar en un parking para personas con discapacidad)	2,37 (0,93)	3,15 (0,91)	4,07 (0,87)	1,67 (1,21)	3,96 (1,29)	2,56 (1,31)	4,22 (0,75)	1,85 (1,29)
**Using many swear words** (Hablar con muchos tacos)	3,83 (1,07)	3,62 (1,35)	3,45 (1,24)	1,76 (0,69)	3,69 (1,20)	2,52 (1,01)	3,56 (1,12)	1,81 (0,99)
**Not saying please when asking for** (No pedir las cosas por favor)	2,73 (0,78)	3,04 (0,90)	3,60 (0,65)	1,89 (0,93)	3,88 (1,21)	1,89 (0,93)	3,67 (0,92)	1,81 (1,69)
**Leaving garbage outside the container** (Poner la basura por fuera del contenedor)	3,38 (0,98)	3,28 (1,00)	2,66 (1,01	1,69 (1,04)	3,55 (0,87)	2,18 (1,09)	3,83 (1,00)	1,81 (1,29)
**Throwing cigarette ashes to the ground** (Tirar las cenizas del cigarro al suelo)	4,52 (1,16)	3,32 (1,35)	2,54 (1,42)	1,88 (0,82)	3,23 (1,24)	2,00 (1,20)	3,08 (1,20)	1,80 (1,00)
**Belching in public** (Eructar en público)	2,25 (1,00)	3,08 (1,09)	3,86 (1,08)	1,93 (1,09)	3,50 (1,17)	1,79 (0,88)	3,04 (0,88)	1,79 (1,03)
**Not using the pedestrian crossing** (No cruzar por el paso de peatones)	3,45 (1,09)	3,59 (1,18)	2,90 (1,21)	1,66 (0,72)	2,45 (1,02)	2,31 (1,11)	4,17 (0,93)	1,76 (1,08)
**Not giving thanks when someone does you a favor** (No dar las gracias cuando alguien te hace un favor)	2,78 (1,05)	3,11 (0,85)	3,44 (0,93)	1,62 (0,75)	4,15 (0,92)	2,22 (1,15)	3,62 (0,98)	1,73 (1,22)
**Emptying your car ashtray onto the street** (Vaciar el cenicero del coche en la calle)	2,66 (1,08)	2,59 (0,78)	3,31 (0,85)	1,45 (0,69)	3,72 (1,22)	2,41 (1,15)	3,76 (1,09)	1,69 (1,32)
**Not respecting the cycle lane** (No respetar el carril bici)	3,52 (1,30)	2,55 (0,83)	3,45 (1,12)	1,76 (0,91)	3,55 (1,24)	2,48 (1,24)	4,21 (1,05)	1,69 (1,58)
**Scratching your genitals ostentatiously** (Rascarse los genitales aparatosamente)	3,10 (1,21)	3,45 (1,09)	3,86 (1,01)	1,59 (0,68)	3,66 (1,01)	1,66 (0,77)	3,07 (1,05)	1,63 (1,27)
**Screaming in public** (Hablar gritando)	3,07 (1,03)	2,57 (0,92)	4,48 (0,57)	1,38 (0,49)	3,43 (1,20)	2,28 (0,88)	4,28 (0,70)	1,62 (1,31)
**Making a lot of noise in public spaces** (Hacer mucho ruido en los espacios públicos)	4,07 (0,94)	3,00 (1,47)	3,93 (1,21)	1,89 (1,07)	3,61 (1,13)	2,32 (1,16)	4,29 (0,85)	1,57 (0,82)
**Pulling up flowers from public gardens** (Arrancar flores de jardines públicos)	2,57 (1,23)	3,07 (1,15)	2,89 (1,31)	1,78 (0,89)	3,68 (1,09)	1,89 (0,83)	3,11 (0,96)	1,54 (1,04)
**Painting graffiti on street furniture** (Hacer pintadas en el mobiliario urbano)	3,00 (1,39)	3,03 (1,02)	3,59 (1,18)	2,03 (0,98)	3,66 (1,17)	2,83 (2,19)	3,83 (0,93)	1,54 (1,27)
**Parking in front of a garage with a no-parking sign** (Aparcar delante de un garaje con vado permanente)	3,00 (1,13)	3,14 (1,06)	4,14 (0,95)	1,55 (0,74)	3,24 (0,99)	2,54 (1,07)	4,33 (0,73)	1,52 (0,91)
**Throwing sunflower seed shells to the ground** (Tirar las cáscaras de las pipas al suelo)	4,14 (1,15)	2,96 (1,23)	2,39 (0,83)	1,82 (0,77)	3,07 (0,98)	1,96 (0,92)	3,07 (1,05)	1,50 (0,85)
**Not offering to help someone in need** (No ofrecer ayuda a quien lo necesita)	2,34 (0,77)	3,48 (0,95)	4,03 (1,05)	1,32 (0,72)	4,31 (1,39)	1,72 (1,39)	4,72 (0,53)	1,48 (1,00)
**Spitting on the street** (Escupir en la calle)	3,79 (1,26)	3,29 (1,27)	3,25 (1,00)	1,68 (0,77)	3,46 (1,32)	1,82 (0,86)	3,14 (1,24)	1,48 (0,88)

**Lying to get something from others** (Mentir para obtener algo de los demás)	3,56 (0,80)	4,33 (0,73)	3,96 (0,81)	1,33 (0,48)	4,41 (1,01)	2,81 (1,47)	4,54 (0,65)	1,48 (1,03)
**Not flushing the toilet in a public washroom** (No tirar de la cadena de un baño público)	3,50 (1,23)	3,18 (1,09)	3,32 (1,22)	1,75 (0,93)	3,36 (1,10)	1,82 (1,06)	3,78 (1,09)	1,46 (0,82)
**Not helping a blind person cross the street** (No ayudar a un invidente a cruzar la calle)	3,14 (1,33)	3,14 (0,99)	3,00 (1,49)	1,79 (0,98)	3,76 (1,33)	2,24 (1,27)	4,34 (0,72)	1,46 (0,81)
**Smoking in a prohibited place** (Fumar en un lugar prohibido)	2,86 (1,22)	3,72 (1,07)	3,83 (1,26)	1,25 (0,59)	3,72 (1,25)	2,41 (1,15)	4,18 (1,09)	1,44 (0,68)
**Not giving your seat up to an older person** (No ceder el asiento a una persona mayor)	3,36 (0,99)	3,25 (1,17)	3,71 (1,15)	1,68 (1,12)	4,46 (0,92)	2,50 (1,20)	4,18 (0,94)	1,43 (0,92)
**Criticizing someone behind their back** (Criticar a alguien a sus espaldas)	4,81 (0,40)	3,81 (1,02)	4,15 (0,95)	1,23 (0,51)	4,58 (0,81)	2,37 (1,47)	4,15 (0,95)	1,41 (0,88)
**Not picking up dog droppings** (No recoger los excrementos del perro)	2,89 (1,28)	3,46 (1,14)	3,79 (0,92)	1,25 (0,44)	3,46 (1,10)	2,00 (1,02)	3,75 (0,93)	1,39 (0,85)
**Throwing chewing gum to the ground** (Tirar el chicle al suelo)	3,56 (1,08)	3,24 (1,20)	2,31 (1,29)	1,46 (0,71)	3,62 (1,10)	1,88 (0,91)	3,38 (1,36)	1,36 (0,92)
**Obstructing traffic by talking to another driver** (Obstaculizar el tráfico hablando con otro conductor)	2,96 (1,32)	2,93 (1,27)	4,50 (0,79)	1,43 (0,92)	3,96 (1,00)	2,89 (2,30)	4,50 (0,75)	1,36 (0,95)
**Ruining the street furniture** (Estropear el mobiliario urbano)	3,11 (1,19)	3,50 (1,14)	3,89 (0,96)	1,21 (0,63)	4,11 (1,01)	1,71 (0,81)	4,18 (0,90)	1,32 (0,88)
**Urinating on the street** (Orinar en la calle)	2,89 (1,03)	3,29 (1,18)	3,61 (1,13)	1,39 (0,57)	3,25 (1,38)	1,50 (0,75)	3,64 (1,10)	1,29 (0,74)
**Hitting another car and leave without taking responsibility** (Dar un golpe a otro coche y marcharse sin responsabilizarse**)**	3,52 (0,98)	2,92 (1,02)	3,85 (1,13)	1,32 (0,48)	4,58 (0,76)	2,26 (1,26)	4,30 (0,61)	1,26 (0,50)
**Ridiculing others for having a different opinion** (Ridiculizar al otro por tener una opinión diferente)	3,48 (0,94)	3,69 (1,12)	4,00 (0,96)	1,12 (0,33)	4,65 (0,69)	2,30 (1,41)	4,52 (0,75)	1,26 (0,55)
**Throwing papers and trash on the street** (Tirar papeles y basura a la calle)	3,32 (1,22)	3,64 (1,28)	3,39 (1,20)	1,21 (0,42)	3,82 (1,09)	1,86 (1,01)	4,00 (0,98)	1,25 (0,71)
**Harassing someone on social networks** (Hostigar a alguien en las redes sociales)	3,78 (1,09)	3,58 (1,24)	3,93 (1,21)	1,12 (0,33)	4,42 (0,81)	2,26 (1,63)	4,56 (0,70)	1,22 (0,59)
**Throwing cigarette butts to the ground** (Tirar las colillas al suelo)	4,34 (1,20)	3,17 (1,07)	2,66 (0,90)	1,52 (0,95)	3,55 (1,27)	2,45 (1,35)	3,62 (1,08)	1,18 (0,81)
**Not apologizing when you bump into someone** (No disculparse al tropezar con alguien)	2,67 (1,14)	2,75 (1,04)	4,07 (1,15)	1,11 (0,42)	4,54 (0,88)	2,00 (1,19)	4,43 (0,79)	1,15 (0,51)
**Disregarding the professional merits of another person** (Despreciar los méritos profesionales de otra persona)	3,59 (1,01)	3,62 (1,02)	3,41 (1,15)	1,15 (0,37)	4,35 (0,89)	2,30 (1,41)	4,30 (1,03)	1,11 (0,37)
**Mocking someone's disability** (Burlarse de la discapacidad de alguien)	2,89 (0,93)	3,15 (0,95)	4,74 (0,59)	1,00 (0,00)	4,48 (1,09)	2,04 (1,37)	4,74 (0,53)	1,07 (0,57)
**Rejoicing in the death of another person** (Alegrarse de la muerte de otra persona)	1,48 (0,51)	3,00 (0,98)	4,63 (0,84)	1,04 (0,19)	4,59 (0,97)	1,96 (1,43)	4,81 (0,62)	1,04 (0,00)

Table S3

Means and standard deviation of each of the clusters, and correlations between dimensions.

	Cluster 1	Cluster 2	1	2	3	4	5	6	7	8
1. Civility	4.27 (0.34)	1.88 (0.50)		-.120	.024	-.473**	.884**	-.694**	.529**	-.704**
2. Frequency	2.86 (0.60)	3.15 (0.69)	.096		.229*	-.229*	-.006	.024	.156	.079
3. Universality	3.52 (0.29)	3.30 (0.36)	.505**	.399**		.086	-.090	-.009	-.062	-.008
4. Censure	2.93 (0.59)	3.39 (0.67)	.401**	.309*	.300		-.593**	.578**	-.087	.662**
5. Valence	4.15 (0.43)	1.84 (0.51)	.781**	.189	.613**	.562**		-.674**	.532**	-.708**
6. Moral information	3.76 (0.58)	3.40 (0.71)	.716**	-.017	.215	.608**	.614**		-.137	.784**
7. Humanness	3.98 (0.33)	2.34 (0.43)	.686**	.225	.448**	.526**	.781**	.694**		-.059
8. Social repercussion	3.58 (0.59)	3.49 (0.78)	.560**	.059	.330*	.755**	.585**	.680**	.600**	

Note. Correlations for civil behaviors (Cluster 1) are below the diagonal and correlations for uncivil behaviors (Cluster 2) are above the diagonal.

Behaviors selection (Study 1)

Two civil behaviors and two uncivil behaviors were selected from a pre-test study of 120 civil and uncivil behaviors evaluated via several dimensions relevant to humanness and civility. The sample used in this pre-test was independent of the main study but the sample has similar characteristics to this one ($N = 360$; $n = 261$ female participants; $M_{age} = 20.01$; $SD = 3.46$; see supplementary materials). Specifically, the two civil behaviors were: "Think about the people who pick up after their dog after it has done its business when out on a walk" and "Think about the people who deposit their glass bottles in the glass recycling bins". The two uncivil behaviors were: "Think about the people who don't use the bike lane" and "Think about the people who leave their garbage out on the street instead of placing it in the bin". To ensure differences in civility and valence for the civil and uncivil behaviors, means were compared for each behavior. The analyses showed differences in civility between civil ($M = 4.15$; $SD = 1.39$) and uncivil behaviors ($M = 1.86$; $SD = 1.22$), $t(115) = 9.48$; $p < .001$; $d = 0.18$, 95% CI [1.81, 2.77], as well as differences in valence ($M_{civil} = 3.89$; $SD_{civil} = 1.52$ and $M_{uncivil} = 1.75$; $SD_{uncivil} = 1.00$) , $t(117) = 9.13$; $p < .001$; $d = 0.17$, 95% CI [1.68, 2.61].

Finally, four behaviors included in the study were unrelated to civility. These two positive and two negative behaviors had been obtained in another pre-test study with 86 behavioral descriptions, in which the sample was independent of the previous pre-test ($N = 64$; $n = 52$ female participants; $M_{age} = 20.50$; $SD = 3.91$). The positive behaviors were "Think about the people who do their grocery shopping" and "Think about the people who tend to go for walks." The negative behaviors were "Think about the people who give their uninformed opinion about anything" and "Think about the people who waste their time instead of making the most of it."

The criteria for selecting civil and uncivil behaviors required they represent high versus low civility and simultaneously be comparable in valence to the behaviors unrelated to civility. An analysis, in which data from both pre-tests was used, verified the civil behaviors were different from the neutral positive behaviors in civility but not in valence. The analyses confirmed the differences in civility: $M = 4.17$, $SD = 0.93$ for the civil behaviors and $M = 3.47$, $SD = 0.66$ for the neutral positive behaviors, $t(29) = 3.00$; $p = .005$; $d = 0.09$, 95% CI [0.22, 1.18]. The analyses also confirmed no difference in valence: $M = 3.88$, $SD = 1.11$ for the civil behaviors and $M = 3.53$, $SD = 0.57$ for the neutral positive behaviors, $t(29) = 1.45$; $p = .157$; $d = 0.04$, 95% CI [0.14, 0.84].

The same analysis was done for the uncivil behaviors and for the neutral negative behaviors. The analyses confirmed the differences in civility: $M = 1.85$, $SD = 0.85$ for the uncivil behaviors and $M = 2.72$, $SD = 0.76$ for the neutral negative behaviors, $t(29) = 3.73$; $p = .001$; $d = 0.11$, 95% CI [1.34, 0.39]. The analyses also confirmed no difference in valence: $M = 1.75$, $SD = 0.64$ for the uncivil behaviors and $M = 1.98$, $SD = 0.44$ for the neutral negative behaviors, $t(29) = 1.68$; $p = .105$; $d = 0.04$, 95% CI [0.52, 0.05].

Traits selection (Study 1)

The traits presented were all positive and represented the four groups defined by Haslam and Bain (2007), resulting from crossing the HN and HU dimensions. The traits were selected from a pretest study that included 144 traits with scores for HN, HU, and valence (*N* = 100; *n* = 70 females; M_{age} = 20.12; *SD* = 3.48). Of these 144 traits, 16 were selected that maximize the differences in high and low scores in the two categories. The 16 traits selected included the high HN–high HU traits passionate, idealistic, imaginative, and rational [apasionado, idealista, imaginativo y racional]; the high HN–low HU traits active, curious, efficient, and emotional [activo, curioso, eficiente y emocional]; the low HN–high HU traits cultured, humble, tolerant, and refined [culto, humilde, tolerante y refinado]; and the low HN–low HU traits uninterested, relaxed, satisfied, and serene [desinteresado, relajado, satisfecho y sereno]. The analysis of the scores for these 16 traits confirmed they adequately represented each category. Thus, the mean scores for traits high (*M* = 4.99 and *SD* =.47) and low (*M* = 3.80 and *SD* =.16) in HN differed significantly (Cohen's *d* = 3.39), *t*(14) = 6.73 and *p* < .001, but not in their mean score on the HU scale, *t*(14) = -.41 and *p* = .686, nor in valence, *t*(14) = 0.84 and *p* = .415. The mean scores for traits high (*M* = 5.68 and *SD* = .57) and low (*M* = 3.15 and *SD* = .63) in HU also differed significantly (Cohen's *d* = 4.21), t(14) = 8.41, *p* < .001, but not on the HN scale, t(14) = .51, *p* = .618, nor in valence, *t*(14) = 0.73, *p* = .478. [1]

1 The traits selected from the pretest study were obtained via the scores that best optimized the four categories of the cross between HN and HU. However, a tautological confirmation could exist because the "refined" trait is, in turn, part of the definition of "civic person" (high HU–low HN category). Thus, once all the analyses had been completed, the trait "refined" was taking out and the analyses of two studies were repeated. The high HU–low HN scores did not differ, and the main studies did not change the pattern of results reported below.

Behaviors selection (Study 2)

The same two civil behaviors and two uncivil behaviors presented in Study 1 were included in Study 2. In the moral condition, two moral behaviors were included: "Think about the people who do not cheat on a test even if they have the answers in front of them" and "Think about the people who stand up for a friend when they are being teased or harassed," whereas in the immoral condition, the following behaviors were used: "Think about the people who cheat on their wife/husband/girlfriend/boyfriend" and "Think about the people who bad-mouth a good friend behind their back." The moral and immoral behaviors were extracted from Chadwick et al. (2006).

A pretest study ($N = 53$) was done to test differences in civility and morality of behaviors. The sample used in this pre-test was independent of the main study. Participants had to indicate (a) the extent to which each behavior was associated with civility (from 1 = *not at all related* to 10 = *extremely related*) and also (b) the extent to which each behavior was associated with morality (from 1 = *not at all related* to 10 = *extremely related*). Two questions were included. The analyses showed that there were differences in civility between civil behaviors, $M = 8.98$, $SD = 1.53$, and moral behaviors, $M = 6.38$, $SD = 2.55$, $t(52) = 7.21$; $p < .001$, and between uncivil behaviors, $M = 6.93$, $SD = 3.00$, and immoral behaviors, $M = 4.24$, $SD = 2.32$, $t(52) = 6.17$; $p < .001$. The same analyses were done for morality, again obtaining differences between civil behaviors, $M = 6.75$, $SD = 2.32$, and moral behaviors, $M = 8.86$, $SD = 1.29$, $t(52) = 5.76$; $p < .001$, and between uncivil behaviors, $M = 4.89$, $SD = 2.50$, and immoral behaviors, $M = 7.08$, $SD = 3.45$, $t(52) = 5.21$; $p < .001$.

Table S4

Descriptive Statistics for Positive and Negative Behaviors Ratings in Humanity Traits in Study 1.

Behavior	High HU		Low HU	
	High HN	Low HN	High HN	Low HN
		Positive behaviors		
Civil	3.31 (.61)$_{a,x}$	3.68 (.53)$_{b,x}$	3.53 (.66)$_{c,x}$	3.15 (.57) $_{a,x}$
Positive	3.17 (.50) $_{a,x}$	3.10 (.40) $_{a,y}$	3.60 (.43) $_{b,x}$	3.09 (.53) $_{a,x}$
		Negative behaviors		
Uncivil	2.31 (.50) $_{a,x}$	1.94 (.55) $_{b,x}$	2.35 (.43) $_{a,x}$	2.78 (.56) $_{c,x}$
Negative	2.54 (.50)$_{a,y}$	2.34 (.47)$_{b,y}$	2.49 (.52) $_{a,x}$	2.96 (.51) $_{c,x}$

Note. Contrast row wise (between traits) are indicated by subscripts a, b, and c. Contrasts column wise (between behavior conditions, for positive and negative separately) are indicated by subscripts x, and y. In each study, for all contrasts, common subscripts indicate the absence of a significant difference at $p < .05$.

Table S5

F ratios resulting from the repeated-measures ANOVA (*Study 1*).

Main Effects and Interactions	*df*	*F*	η^2_p
Human Uniqueness	1,188	62.97***	.25
Human Uniqueness*Type of behavior	1,188	4.81*	.03
Human Uniqueness*Valence	1,188	47.37***	.20
Human Uniqueness*Type of behaviour*Valence	1,188	27.02***	.13
Human Nature*Valence	1,188	13.25**	.07
Human Nature*Type of behavior*Valence	1,188	9.17**	.05
Human Nature*Human Uniqueness*Valence	1,188	137.63***	.42
Human Nature*Human Uniqueness*Type of behavior*Valence	1,188	3.96*	.02

Note. *$p < .05$; **$p < .01$; ***$p < .001$

Table S6

Descriptive Statistics for Positive and Negative Behaviors Ratings in Humanity Traits in Study 2.

Behavior	High HU		Low HU	
	High HN	Low HN	High HN	Low HN
		Positive behaviors		
Civil	3.40 (.48) $_{a,x}$	3.65 (.51) $_{b,x}$	3.52 (.45) $_{a,x}$	3.16 (.65) $_{c,x}$
Moral	3.51 (.65) $_{a,x}$	3.48 (.58) $_{a,x}$	3.57 (.55) $_{a,x}$	3.15 (.56) $_{b,x}$
Positive	3.14 (.46) $_{a,y}$	3.14 (.43) $_{a,y}$	3.56 (.45) $_{b,x}$	3.15 (.56) $_{a,x}$
		Negative behaviors		
Uncivil	2.05 (.64) $_{a,x}$	1.92 (.59) $_{b,x}$	2.25 (.60) $_{c,x}$	2.83 (.58) $_{d,x}$
Immoral	2.73 (.56) $_{a,y}$	2.10 (.37) $_{b,x}$	2.92 (.56) $_{c,y}$	2.46 (.65) $_{d,y}$
Negative	2.73 (.41) $_{a,y}$	2.41 (.40) $_{b,y}$	2.55 (.48) $_{c,z}$	3.00 (.48) $_{d,x}$

Note. Contrast row wise (between traits) are indicated by subscripts a, b, c, and d. Contrasts column wise (between behavior conditions for positive and negative separately) are indicated by subscripts x, y, and z. In each study, for all contrasts, common subscripts indicate the absence of a significant difference at $p < .05$.

Table S7

F ratios resulting from the repeated-measures ANOVA (*Study 2*).

Main Effects and Interactions	*df*	*F*	η^2_p
Human Uniqueness	1,322	57.03***	.15
Human Uniqueness*Type of behavior	2,322	4.72*	.03
Human Uniqueness*Valence	1,322	85.172***	.21
Human Uniqueness*Type of behaviour*Valence	2,322	29.234***	.15
Human Nature	1,322	25.458***	.07
Human Nature*Type of behavior	2,322	32.31***	.17
Human Nature*Type of behaviour*Valence	2,322	17.25***	.10
Human Nature*Human Uniqueness*Type of behavior	2,322	3.66*	.02
Human Nature*Human Uniqueness*Valence	1,322	127.89***	.28
Human Nature*Human Uniqueness*Type of behavior*Valence	2,322	7.10**	.04

Note. *$p < .05$; **$p < .01$; ***$p < .001$
